# Role of magnesium supplementation in the treatment of depression: A randomized clinical trial

**DOI:** 10.1371/journal.pone.0180067

**Published:** 2017-06-27

**Authors:** Emily K. Tarleton, Benjamin Littenberg, Charles D. MacLean, Amanda G. Kennedy, Christopher Daley

**Affiliations:** 1 Center for Clinical and Translational Science, University of Vermont, Burlington, Vermont, United States of America; 2 Department of Medicine, University of Vermont, Burlington, Vermont, United States of America; 3 Department of Psychiatry, University of Vermont, Burlington, Vermont, United States of America; Indiana University Richard M Fairbanks School of Public Health, UNITED STATES

## Abstract

Current treatment options for depression are limited by efficacy, cost, availability, side effects, and acceptability to patients. Several studies have looked at the association between magnesium and depression, yet its role in symptom management is unclear. The objective of this trial was to test whether supplementation with over-the-counter magnesium chloride improves symptoms of depression. An open-label, blocked, randomized, cross-over trial was carried out in outpatient primary care clinics on 126 adults (mean age 52; 38% male) diagnosed with and currently experiencing mild-to-moderate symptoms with Patient Health Questionnaire-9 (PHQ-9) scores of 5–19. The intervention was 6 weeks of active treatment (248 mg of elemental magnesium per day) compared to 6 weeks of control (no treatment). Assessments of depression symptoms were completed at bi-weekly phone calls. The primary outcome was the net difference in the change in depression symptoms from baseline to the end of each treatment period. Secondary outcomes included changes in anxiety symptoms as well as adherence to the supplement regimen, appearance of adverse effects, and intention to use magnesium supplements in the future. Between June 2015 and May 2016, 112 participants provided analyzable data. Consumption of magnesium chloride for 6 weeks resulted in a clinically significant net improvement in PHQ-9 scores of -6.0 points (CI -7.9, -4.2; *P*<0.001) and net improvement in Generalized Anxiety Disorders-7 scores of -4.5 points (CI -6.6, -2.4; *P*<0.001). Average adherence was 83% by pill count. The supplements were well tolerated and 61% of participants reported they would use magnesium in the future. Similar effects were observed regardless of age, gender, baseline severity of depression, baseline magnesium level, or use of antidepressant treatments. Effects were observed within two weeks. Magnesium is effective for mild-to-moderate depression in adults. It works quickly and is well tolerated without the need for close monitoring for toxicity.

## Introduction

Depression affects 350 million people worldwide and is predicted to be the leading cause of disease burden by 2030, based on disability-adjusted-life-year [1]. Initial antidepressant trials of adequate dose and duration result in only about 50% of patients achieving remission [2]. Even after the addition of other treatments, 20% still suffer from symptoms after 2 years. Non-pharmacologic approaches such as Cognitive Behavioral Therapy and lifestyle interventions require highly trained therapists and several weeks to months to achieve effectiveness [3]. There is a great need for additional treatment options.

The association between magnesium intake and depression is well documented [4–7]. Improvement in depression with magnesium supplementation has been reported inconsistently [8, 9], although few clinical trials exist. One trial found magnesium chloride to be effective for depression in seniors with type 2 diabetes [10] while another trial found magnesium citrate decreased depression in patients with fibromyalgia [11]. One negative trial used magnesium oxide [12], known to be poorly absorbed.

The aim of this study was to test the hypothesis that 6 weeks of oral magnesium chloride (MgCl_2_) supplementation will improve symptoms of mild-to-moderate depression in a primary care population.

## Methods

### Trial design

This was a 12-week open label randomized cross-over control trial. Participants were recruited through primary care providers (PCPs) within a single academic medical center and randomized to begin MgCl_2_ supplementation immediately or at week 7 (delayed). During the other 6-week period, they took no MgCl_2_. Prior to the start of the study the Institutional Review Board of the University of Vermont approved the study. All subjects provided written informed consent. Trial registry can be found at clinicatrials.gov (Identifier: 02466087).

### Participants

The target population was adults with mild-to-moderate depression. Inclusion criteria were: 1) 18 years of age or older; 2) no change in treatment plan for depression for the past 2 months and going forward (including no current treatment, stable use of antidepressant medication, or ongoing nonpharmacologic therapy); 3) Patient Health Questionnaire-9 (PHQ-9) score of 5–19 [13]. Exclusion criteria were: 1) Schizophrenia, bipolar disease, active delirium, dementia, kidney disease (due to the role of the kidneys in magnesium homeostasis), myasthenia gravis (magnesium may worsen symptoms of the disease), or gastrointestinal (GI) disease (diarrhea is a common side effect of magnesium); 2) pregnant or trying to get pregnant; 3) planned surgery in the next 3 months; 5) taking a medication known to interact with magnesium; 6) unwilling to stop taking non-study magnesium supplements for the duration of the study.

### Magnesium supplements

Tablets of MgCl_2_ (Alta Health Products, Idaho City, ID) were provided free of charge. Participants were instructed to take four 500 mg tablets of magnesium chloride daily for a total of 248 mg of elemental magnesium per day. MgCl_2_ was used because of its high bioavailability and tolerability compared to other salts [14, 15].

### Study procedures and randomization

PCPs reviewed lists of their patients with a diagnosis of depression in their medical record and indicated which ones may be sent a letter describing the study. PCPs were encouraged to remove patients from their list if they knew depression was no longer an active problem, the patient was also suffering from severe mental illness, or the patient was not able to start or stop taking magnesium. Those patients that did not opt out after receiving the letter were contacted by phone to determine interest and eligibility. Eligibility and diagnosis of depression was confirmed with an initial telephone PHQ-9 score between 5 and 19. Participants next met with study staff for a baseline visit during which they provided written informed consent and baseline data including demographics, medication use, the PHQ-9 [13], the Generalized Anxiety Disorders-7 (GAD-7) [16], the Modified Morisky Scale [17] to assess medication adherence behavior, the PhenX Tobacco Smoking Status Questionnaire for Adults, and the PhenX Alcohol 30 Day Quantity and Frequency Questionnaire [18]. Randomization to Immediate and Delayed treatment was stratified based on PHQ-9 score (5–9, 10–14, and 15–19) and blocked in groups of 10. Treatment assignments were sealed in an opaque envelope and shuffled and then numbered and opened in that order. The principal investigator (PI) assigned the participants to their randomization order. The PI also gave the volunteers the supplements at either week 1 or week 7, based on randomization, and educated each participant on the dosage and possible side effects. Data were collected every 2 weeks via telephone and included the PHQ-9, GAD-7, questions about changes in medications, changes in treatment for depression, and side effects.

### Outcome measures

The primary hypothesis was that magnesium supplementation decreases symptoms of depression and therefore the primary outcome was the difference in the change in PHQ-9 scores between baseline and the end of each six-week period (difference in differences). The PHQ-9 is a validated questionnaire with high sensitivity and specificity for the diagnosis of depression [13]. The PHQ-9 score can range from 0 to 27, with the following severity scores: 0–4 None; 5–9 Mild; 10–14 Moderate; 15–19 Moderate to Severe; 20–27 Severe. Telephone administration is comparable to in-person tracking [19].

Secondary outcomes were exploratory and included changes in the GAD-7 score as well as adherence to the supplement regimen and intention to use magnesium supplements in the future. GAD-7 score was recorded in the same fashion as the PHQ-9 and has been shown to be a valid indication of anxiety symptoms [16]. The GAD-7 score can range from 0 to 21, with the following severity scores: 0–4 None; 5–9 Mild; 10–14 Moderate; 15–21 Severe. To assess side effects, participants were asked to compare symptoms (headache, diarrhea, nausea, constipation, dizziness, oliguria, and polyuria) to baseline using a standardized 0–4 point scale (none, mild, moderate, or severe). At the end of week 12, a pill count was used to calculate adherence to the supplement regimen and participants were asked whether they planned to continue using magnesium and why.

### Data analysis

All data were analyzed based on the intention-to-treat principle. The age and gender of patients who were contacted but ineligible were compared to those who were randomized. Baseline characteristics of eligible participants were compared by randomization group. Student t-tests or Wilcoxon Rank Sum tests were used for continuous values and Chi-square tests for categorical values.

The change in outcome for each patient was calculated as the last value measured during that treatment arm minus the last value measured before that treatment arm. Before crossing over, this was the week 6 measure minus the baseline measure. After cross-over, this was the week 12 measure minus the week 6 measure. If a week 12 measure was not available, the week 10 or week 8 measure was used. Participants who did not provide at least one outcome measure in each treatment period were excluded. Treatment efficacy was assessed as the net improvement in outcome. The mean change in the outcomes during the 6 weeks of the control (no treatment) period was compared to the change in scores during the 6 weeks of treatment. Linear regression was used to test the significance in the net improvement in the outcome while controlling for potential confounders.

Each potential confounder was tested in a separate univariate linear regression for association (*P*<0.05) with the primary outcome and secondary outcome. Potential confounders were included in multivariate models. We explored the effectiveness of treatment among various subgroups using multivariate models. Linear regression adjusting for randomization and clustering was used to identify adverse effects. Cuzick’s test of trend [20] was used to explore the relationships between both the Modified Morisky score and treatment response with adherence. A two-sided *P*<0.05 was considered statistically significant. All analyses were completed using Stata14·1 (College Station, TX).

The targeted sample size was based on detection of a difference in difference in PHQ-9 scores of 1.5, which was felt to be clinically significant. The calculation, assuming a paired t-test, with 84% power, type I error rate of 5%, and a standard deviation of 5 [21], resulted in a sample size of 50 participants in each group.

## Results

**Fig 1:**
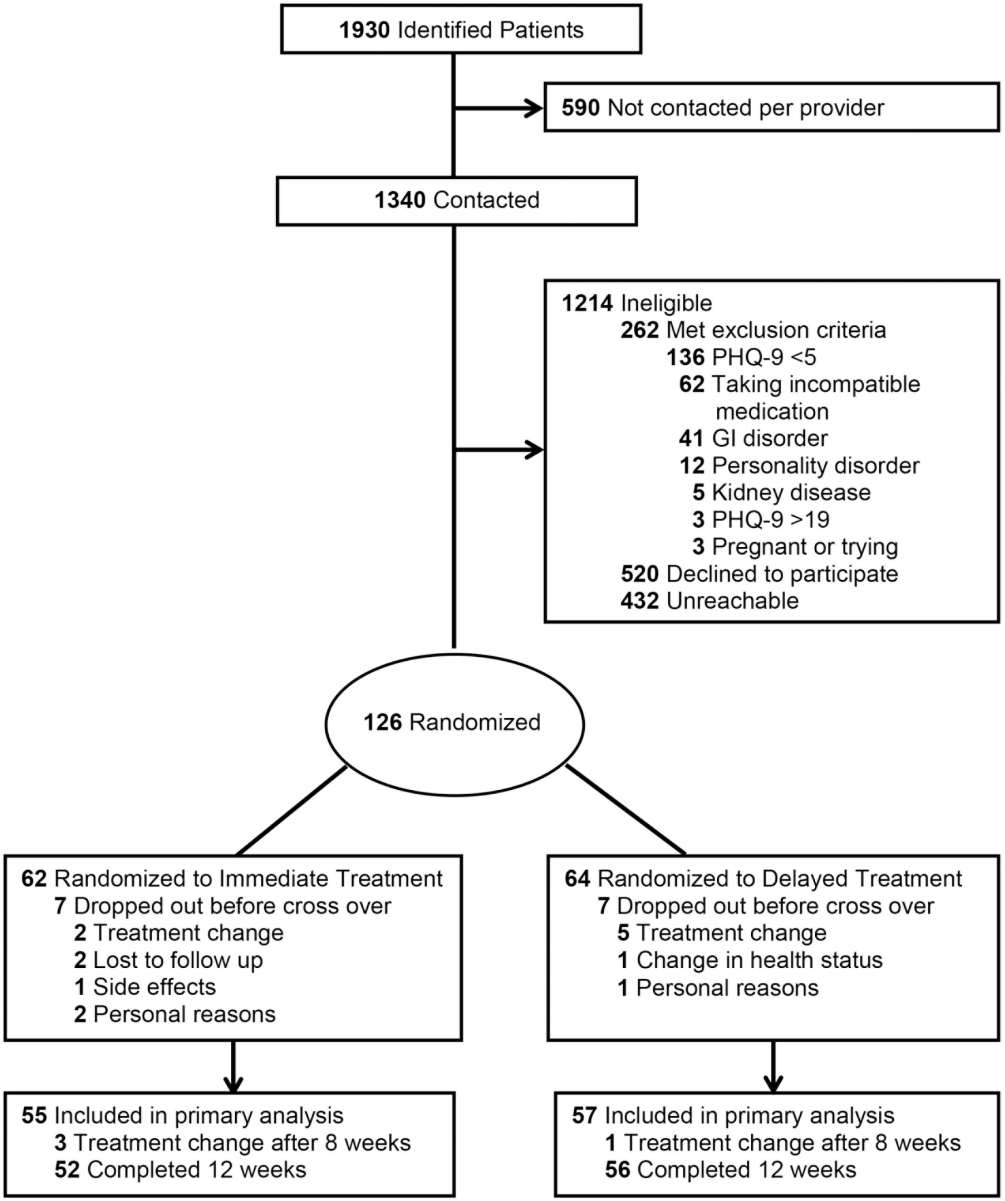
Consort diagram.

Recruitment occurred between June 2015 and May 2016. Of 1,930 patients identified from medical records, 1,340 (68%) were contacted and 126 (7%) were eligible and randomized (Fig 1). The mean age of the contacted patients was 50 years compared to a mean age of 52 years in the randomized group (*P* = 0.06). The randomized group had fewer males than the other contacted patients (38% *vs*.47%; *P* = 0.07).

Sixty-two participants (49%) were randomized to Immediate Treatment and 64 (51%) to Delayed Treatment (*P* = 0.95). The two groups were similar in all baseline characteristics except age. The mean age in the Immediate group was 55.6 versus 49.1 in the Delayed group (*P* = 0.006). All participants commenced treatment based on allocation. Seven participants withdrew from each group before crossing over (11%) (Fig 1). The most common reason was a change in other depression treatment (n = 7) (Table 1). No participants withdrew due to non-compliance. 108 participants completed all 12 weeks of the study. Four withdrew between week 8 and 12; their last results recorded before withdrawal were included in the final sample, resulting in 112 participants analyzed (Fig 1). The characteristics of the final analyzed sample appear in Table 2. The Immediate group was similar to the Delayed group except that they were 5.1 years older (*P* = 0.04).

**Table 1:** Withdrawals by reason and time point.

*Reason for withdrawal*	*Week 2*	*Week 4*	*Week 6*	⨉	*Week 8*	*Week 10*	*Week 12*	*Total*
Treatment Change for Depression	3	3	1	Cross-over		2^a^	2^a^	11
Side Effects	1							1
Change in Health Status		1						1
Personal Reasons			1					1
No Reason Given		1	1					2
Lost to Follow Up	1		1					2
Total number of withdrawals	5	5	4		0	2^a^	2^a^	18

^a^These participants were included in the final analysis using their last recorded values.

**Table 2:** Demographic characteristics of final sample (N = 112).

*Characteristic*	*Randomization Group*		*P-Value*
	*Immediate (N = 55)*	*Delayed (N = 57)*	
Age, mean (SD)	55.2 (12.3)	50.1 (13.0)	0.038
Male Gender, N (%)	22 (40%)	22 (36%)	>0.99
Self-Report White Race, N (%)	53 (96%)	56 (98%)	0.62
Current Smoker, N (%)	7 (13%)	8 (12%)	>0.99
Servings of Alcohol Per Week, mean (SD)	3.3 (5.0)	4.9 (7.8)	0.19
Current Treatment for Depression, N (%)			
No Treatment	14 (25%)	17 (30%)	0.68
Self-management	1 (2%)	1 (2%)	>0.99
Non-pharmacologic Therapy	14 (26%)	11(19%)	0.50
One or more medications	35 (64%)	35 (61%)	0.85
Selective Serotonin Reuptake Inhibitors	19 (35%)	22 (39%)	0.70
Selective Norepinephrine Reuptake Inhibitors	8 (15%)	8 (14%)	>0.99
Tricyclic	2 (4%)	1 (2%)	0.61
Bupropion	7 (13%)	9 (16%)	0.80
Monoamine Oxidase Inhibitors	0	0	-
Antipsychotic	0	2 (4%)	0.50
Baseline Patient Health Questionnaire-9 Depression Score, mean (SD)	10.7 (3.7)	10.6 (3.8)	0.84
Baseline Generalized Anxiety Disorder-7 Anxiety Score, mean (SD)	8.6 (5.1)	8.7 (5.4)	0.92
Modified Morisky Score, mean (SD)	2.9 (0.9)	2.9 (1.0)	0.91

N = number; SD = standard deviation.

*P*-values calculated by Chi-square for categorical values and two-sample t-test or Wilcoxon Rank Sum for continuous values.

The characteristics of the 14 subjects who withdrew before crossing over were similar in all measured characteristics to the 112 in the final sample except that they were more anxious (GAD-7 12.5 *vs*. 8.6; *P* = 0.01). There were no significant differences in age, gender, race, smoking, alcohol consumption, baseline PHQ-9 score, Modified Morisky score, or use of depression therapies at the time of randomization.

### Outcomes

**Fig 2:**
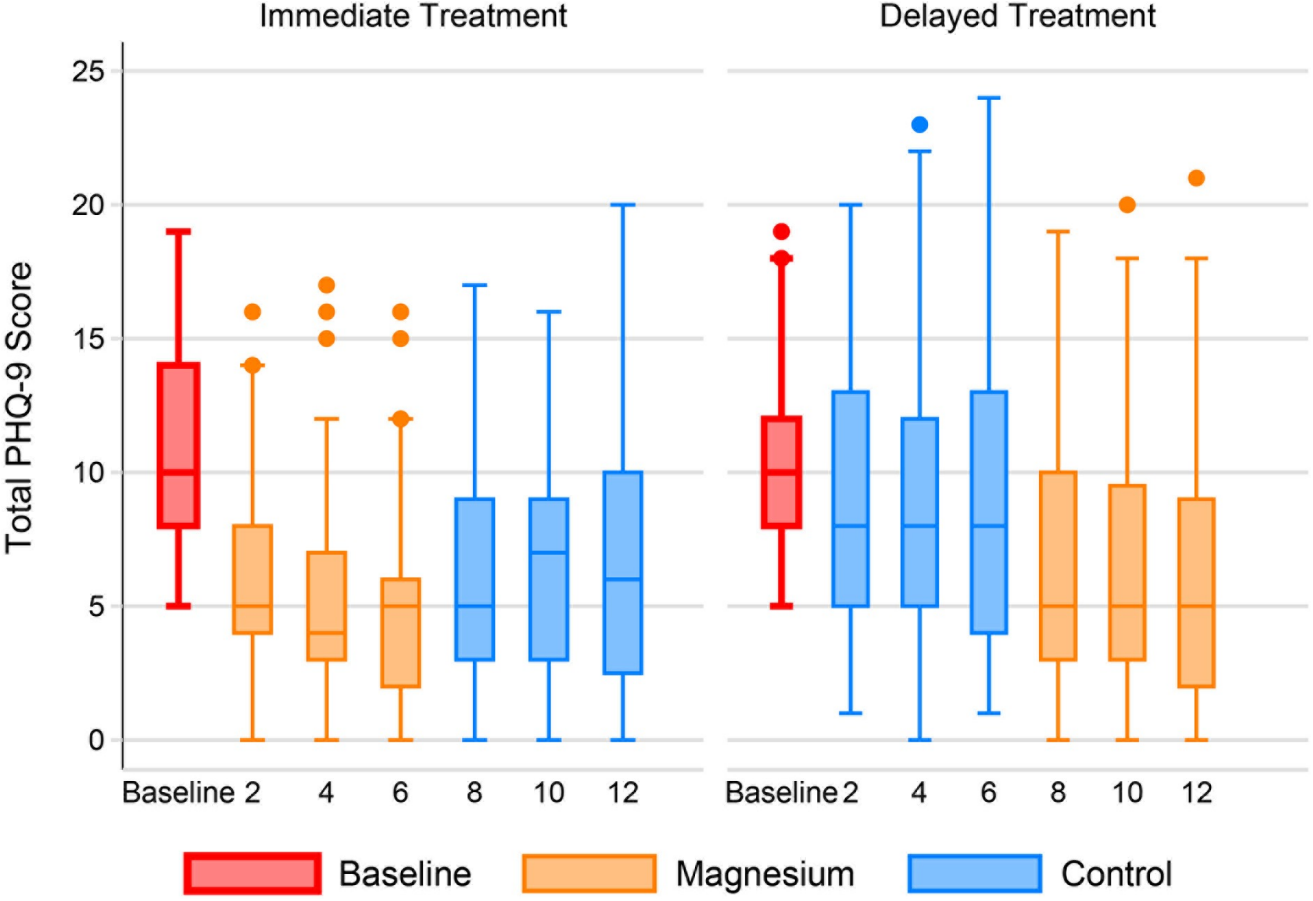
Patient Health Questionnaire-9 over time by group. The individual box plots show the distribution of PHQ-9 scores by week in each randomization group. The middle line of each box represents the median score. The boxes range from the 25^th^ to the 75^th^ percentile. The whiskers demonstrate the range of scores with outliers shown by small circles.

Unadjusted PHQ-9 depression scores improved during magnesium treatment (-4.3 points; 95% confidence interval (CI) -5.0, -3.6), but not during the control period (-0.1; CI -0.9, +0.7) for the final analyzable cohort of 112 adults. The net improvement was -4.2 points (CI -5.4, -2.9; *P<*0.001). Participants who were randomized to Immediate Treatment first experienced a decrease in PHQ-9 score within 2 weeks; their scores increased slightly towards baseline during the 6 weeks of control (Fig 2). Those in the Delayed Treatment group experienced a slight improvement in PHQ-9 score during the control weeks and a further improvement during the active treatment.

Age, gender, race, smoking status, drinks of alcohol per week, adherence to the supplement regimen, and other treatments for depression were not associated with response to treatment and were not included in the adjusted model. Mean PHQ-9 change during the control weeks, randomization order, and use of selective serotonin reuptake inhibitors (SSRI) were retained in the multivariate analyses. When adjusted for these potential confounders, the net improvement with supplementation was -6.0 points (CI -7.9, -4.2; *P*<0.001). See Table 3. Data for all participants (N = 126) follow a similar pattern (Table 4).

**Table 3:** Adjusted net improvement^a^ with magnesium.

			*PHQ-9*			*GAD-7*		
		*N*	*Change*	*95% CI*	*P*	*Change*	*95% CI*	*P*
All subjects	Magnesium	112	-4.9	-6.0, -3.9	<0.001	-3.6	-4.9, -2.3	<0.001
	Control	112	+1.1	-0.1, +2.3	0.08	+0.9	-0.4, +2.1	0.17
Net Improvement			-6.0	-7.9, -4.2	<0.001	-4.5	-6.6, -2.4	<0.001
*Subgroups*			*Net Improvement*	*95% CI*	*P*	*Net Improvement*	*95% CI*	*P*
Gender	Female	68	-6.6	-9.1, -4.0	<0.001	-3.8	-6.4, -1.1	0.003
	Male	44	-5.3	-7.6, -3.1	<0.001	-5.5	-8.9, -2.1	0.001
Age	≤55 years	55	-5.3	-7.9, -2.8	<0.001	-5.1	-8.6, -1.5	0.002
	>55 years	57	-6.5	-9.0, -4.1	<0.001	-4.0	-6.6, -1.5	0.001
Baseline PHQ-9	≤9	49	-4.7	-6.3, -3.2	<0.001	-3.1	-4.8, -1.3	<0.001
	>9	63	-7.2	-10.1, -4.2	<0.001	-5.6	-9.2, -2.1	0.001
Baseline GAD-7	≤9	68	-4.7	-6.8, -2.6	<0.001	-2.2	-4.0, -0.5	0.005
	>9	44	-8.2	-11.0, -5.3	<0.001	-8.3	-12.6, -3.9	<0.001
Adherence	Low	56	-5.3	-8.2, -2.5	<0.001	-3.3	-5.9, -0.6	0.008
	High	56	-6.6	-8.7, -4.6	<0.001	-5.7	-8.7, -2.7	<0.001

PHQ-9 = Patient Health Questionnaire-9; GAD-7 = Generalized Anxiety Disorder-7; CI = confidence interval.

^a^Net Improvement = change in outcome during magnesium treatment–change in outcome during control.

All results adjusted for mean PHQ-9 score during control weeks, treatment order (Immediate *vs*. Delayed), and selective serotonin reuptake inhibitor (SSRI) therapy.

**Table 4:** Unadjusted PHQ-9 and GAD-7 scores by event for all participants (N = 126).

* *	*Patient Health Questionnaire-9 *			*Generalized Anxiety Disorder-7*		
* *	*Randomized Treatment Assignment*		*Total*	*Randomized Treatment Assignment*		*Total*
*Event*	*Immediate*	*Delayed*		*Immediate*	*Delayed*	
Baseline, mean	10.9	10.8	10.8	8.9	9.2	9.0
SD	3.8	3.9	3.8	5.1	5.6	5.3
N	62	64	126	62	64	126
Week 2, mean	7.0	9.1	8.1	5.7	8.3	7.0
SD	4.7	4.9	4.9	4.7	5.4	5.2
N	60	63	123	60	63	123
Week 4, mean	5.8	8.9	7.4	4.9	7.8	6.4
SD	4.3	5.4	5.1	4.4	5.6	5.2
N	59	61	120	59	61	120
Week 6, mean	5.1	9.2	7.1	4.4	9.2	6.8
SD	3.9	5.6	5.2	4.0	5.9	5.6
N	57	57	114	57	57	114
Week 8, mean	6.1	6.8	6.5	5.5	6.2	5.9
SD	4.4	4.9	4.6	4.5	5.5	5.0
N	55	57	112	55	57	112
Week 10, mean	6.5	6.6	6.5	5.3	5.8	5.5
SD	3.9	4.5	4.2	4.3	5.4	4.9
N	52	56	108	52	56	108
Week 12, mean	6.3	6.3	6.3	5.2	5.8	5.5
SD	4.6	5.4	5.0	4.9	5.8	5.3
N	52	56	108	52	56	108

SD = standard deviation; N = number

**Fig 3:**
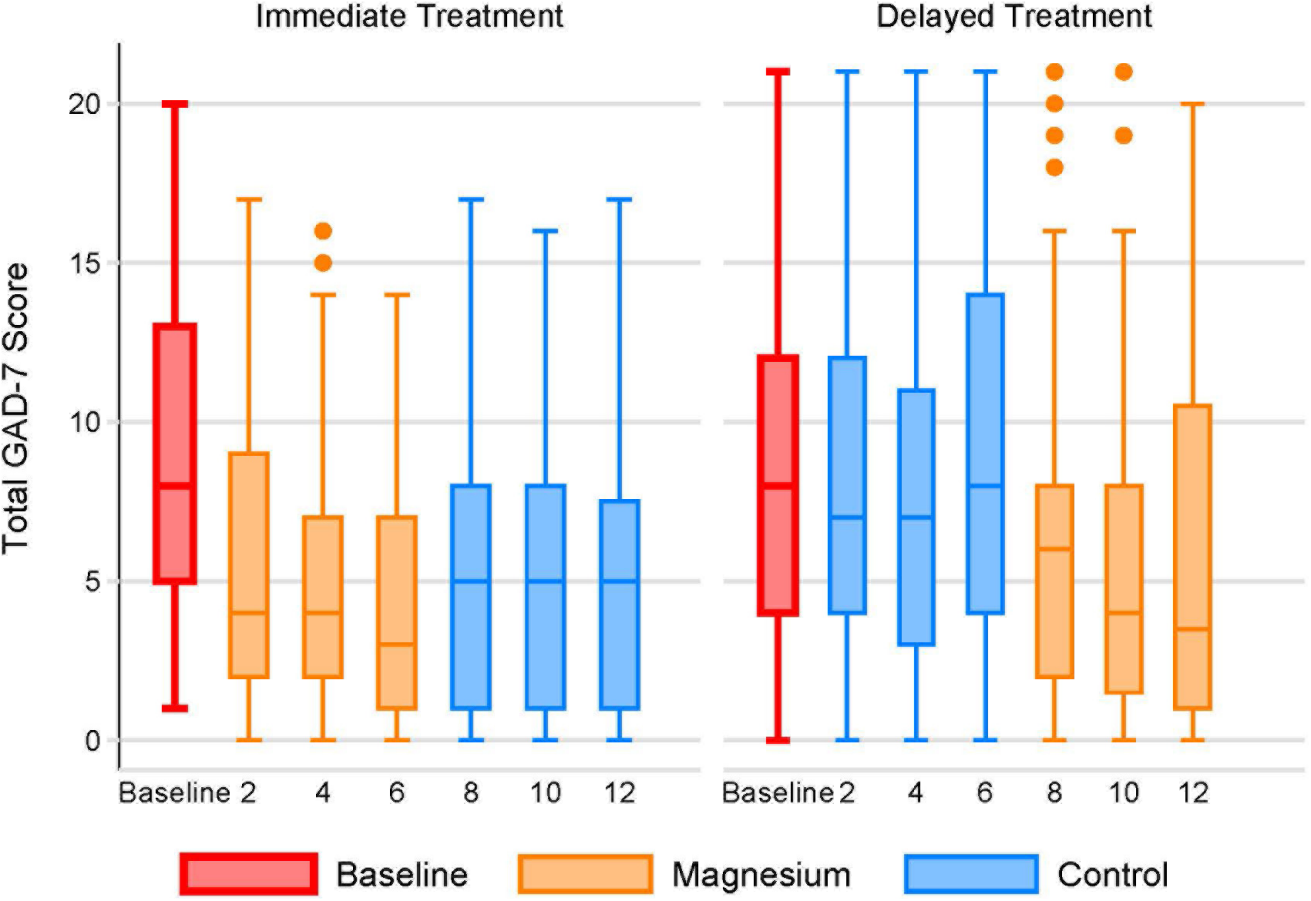
Generalized Anxiety Disorders-7 over time by group. The individual box plots show the distribution of PHQ-9 scores by week in each randomization group. The middle line of each box represents the median score. The boxes range from the 25^th^ to the 75^th^ percentile. The whiskers demonstrate the range of scores with outliers shown by small circles.

Unadjusted GAD-7 anxiety scores improved during magnesium supplementation (-3.9 points; CI -4.7, -3.1), but worsened during the control period (+0.8; CI +0.02, +1.6) for a net benefit of -4·7 points (CI -6.0, -3.3; *P*<0.001) (Fig 3). After adjustment for potential confounders (Table 3), the net improvement in anxiety with magnesium supplementation was -4.5 points (CI -6.6, -2.4; *P*<0.001). Again, the data are similar for all participants as well (Table 4).

Subgroup analyses were performed using the adjusted models of the association of magnesium with PHQ-9 and GAD-7 scores. Subgroups were defined by gender, age above or below 55, PHQ above or below 9, GAD above or below 9, use of any antidepressant medications, use of specific medications (SSRIs, selective norepinephrine reuptake inhibitors, bupropion, monoamine oxidase inhibitors, antipsychotics), use of behavioral therapy or counseling, and adherence above or below 80% by pill count. The analyses indicated that magnesium was effective in all subgroups (Table 3).

Participants were less likely to report headaches while taking magnesium compared to the control period (unadjusted mean headache score 0.41 *vs*. 0.57 on the 0–3 scale). The adjusted difference was -0.16 (CI -0.25, -0.03; *P* = 0.013). There was no difference in the reporting of diarrhea, constipation, nausea, dizziness or urinary symptoms (Table 5).

**Table 5:** Adverse effects during treatment^a^.

	*Unadjusted*			*Adjusted*^b^		
*Adverse effect*	*Control*	*Magnesium*	*Difference*	*Difference*	*95% CI*	*P*
Headache	0.57	0.41	-0.16	-0.14	-0.25, -0.03	0.01
Diarrhea	0.32	0.29	-0.02	-0.01	-0.11, +0.08	0.79
Nausea	0.22	0.24	+0.02	+0.02	-0.07, +0.11	0.64
Constipation	0.20	0.20	+0.00	-0.00	-0.08, +0.07	0.97
Dizziness	0.24	0.22	-0.02	-0.02	-0.09, +0.06	0.66
Oliguria	0.04	0.07	+0.03	+0.03	-0.02, +0.08	0.19
Polyuria	0.11	0.16	+0.05	+0.05	-0.01, +0.11	0.09

CI = confidence interval.

^a^Mean values of biweekly reports on a 0 to 4 scale.

^b^Adjusted for mean PHQ-9 score during control weeks, treatment order (Immediate *vs*. Delayed), use of SSRIs, and clustering within participant.

Using the adjusted model, we explored the effect of magnesium supplementation on the answers to individual PHQ-9 and GAD-7 items. All items in the PHQ-9 improved significantly during active treatment except question 8 (abnormal movement speed) and question 9 (thoughts of suicide). Of note, question 9 was positive on only 3 of 892 occasions. The only GAD-7 questions that did not improve significantly were questions 1 (feeling nervous, anxious, or on edge) and 5 (experiencing restlessness).

Percent adherence in the Immediate Treatment group (83%) and Delayed Treatment group (82%) were similar (*P* = 0·85). Treatment response for both the PHQ-9 and GAD-7 tended to be greater with increased adherence; however, the trend was not significant for either at *P* = 0.19 and *P* = 0.64, respectively.

When asked whether they would take magnesium in the future, 68 (61%) answered yes, 22 (20%) answered maybe and 22 (20%) answered no. The most common reasons for a positive answer were “the magnesium helped my mood” (58%) and “it helped in other ways” (23%), such as by increasing energy, decreasing constipation, and decreasing muscle aches and cramps. The most common reason for a negative response was that “magnesium did not help mood” (46%), followed by side effects (20%). The most common side effect, diarrhea, was reported by 8 participants.

## Discussion

This trial was conducted to test the efficacy and safety of over-the-counter magnesium and to determine its role in the treatment of depression. Consumption of 248 mg of elemental MgCl_2_ daily for 6 weeks improved depression scores by a statistically and clinically significant mean of 6 points and anxiety by over 4 points. This effect was not due to natural history, regression to the mean, or confounding, and was seen in a wide range of patients with varying ages, co-treatments, and severity of baseline symptoms. The similar effects seen in the univariate and multivariate models indicates that the potential confounders had little impact on the estimates of treatment effect.

As with other studies, [8, 11, 22] the improvement in symptoms was seen within weeks. The effect was somewhat diminished within 2 weeks of stopping supplementation, indicating relatively quick clearance as well. Although females are more likely to be diagnosed with depression [23], there was no difference in effect based on gender. The finding that high and low adherence subgroups had similar improvement suggests that a smaller dose may suffice with less risk for side effects and lower cost.

Adverse effects were not so severe as to lead to discontinuation except in one case in which nausea and lethargy led to withdrawal after two weeks. Participants did report experiencing other clinically significant, and well documented, positive effects of taking magnesium, such as decreases in headaches and muscle cramps [24]. The fact that nearly all specific PHQ-9 and GAD-7 items improved significantly while on treatment corresponds with the qualitative reports.

Although the association between magnesium and depression is well documented, the mechanism is unknown. However, magnesium plays a role in many of the pathways, enzymes, hormones, and neurotransmitters involved in mood regulation [25]. It is a calcium antagonist and voltage-dependent blocker of the *N*-methyl-D-aspartate channel which regulates the flow of calcium into the neuron [26]. In low magnesium states, high levels of calcium and glutamate may deregulate synaptic function, resulting in depression [9]. Depression and magnesium are also both associated with systemic inflammation [27, 28]. The finding that those participants taking an SSRI experienced an even greater positive effect points toward magnesium’s possible role in augmenting the effect of antidepressants. Since the mechanism of magnesium’s role in depression is still not clear, it is difficult to say why this relationship with antidepressants may exist. In a sample of treatment resistant depressed patients with normal magnesium levels, those with high normal magnesium levels had a more robust response to antidepressants [29]. In another study, severity of depression correlated with reduced intracellular magnesium, and that cellular levels normalized after successful treatment with antidepressants [30]. Patients may have normal plasma concentration of magnesium yet have depleted intracellular stores [31]. There may also be differential effects for SSRIs compared to other antidepressants. Some evidence to support adjunctive use of other nutraceuticals with antidepressants exists. The mechanism may be related to their anti-inflammatory properties or role in NMDA and glutamate activity [32]. Magnesium supplementation may allow for lower antidepressant dosage or avoid the need for use of a second medication, both of which could reduce overall side effect burden.

### Implications for practice

The net improvement in PHQ-9 score of 6 points is statistically and clinically significant. A change in score of 5 or greater reflects a clinically relevant change in individuals receiving depression treatment. After 6 weeks of psychological counseling, a drop in 5 points from baseline PHQ-9 indicates the treatment response is adequate and no treatment change is needed. The same guidelines can be applied to 4 weeks of an adequate dose of an antidepressant.[33] Magnesium supplementation provides a safe, fast and inexpensive approach to controlling depressive symptoms. Most patients who experience improvement do so within two weeks of starting supplements. Oral magnesium supplementation is safe in adults with normal kidney function who are not taking medications that interact with the supplement and when used in dosages below the upper tolerable limit set by the Institute of Medicine (350 mg elemental magnesium per day) [34]. Hypermagnesemia is most commonly associated with the combination of impaired renal function and excessive intake of nonfood magnesium; few serious adverse effects are reported until very high doses are ingested [34].

Similar to national surveys [35], some participants with depression were not on any treatment. There are many barriers to treatment for depression including stigma associated with diagnosis, cost, side effects, non-adherence, and loss to follow-up [36]. Magnesium supplements do not come with the added stigma associated with other therapies and, while monitoring response is still important, the risk of side effects is not as great as from antidepressants. Over-the-counter magnesium can be offered as an alternative therapy to those patients hesitant to begin antidepressant treatment and is easily accessible without a prescription for around $14.00 per month.

### Strengths

This is the first clinical trial done on magnesium for depression in the U.S. Exclusion criteria were minimal, increasing generalizability, and it used a well-absorbed form of magnesium. The paired analysis allowed each subject to serve as his or her own control, minimizing variance and improving statistical power. Random assignment of treatment order allowed for controlling for regression to the mean as an explanation for the apparent treatment effects. Enrolling patients over a full year minimized the effects of seasonal changes in depression. The withdrawal rate was low and adherence was high, confirming patient reports of high acceptability.

### Limitations

There was no placebo arm and randomization was not blinded for either the study team or the volunteer. The use of placebo and blinding are essential for a study that seeks to understand the mechanism of action of an intervention. However, they are not useful when the research seeks to assess the presence and magnitude of the effect of an intervention. Whether magnesium works because it induces a physiological change in the subject, or only because of the placebo effect (or a combination of the two), it remains that subjects do report better levels of depression and anxiety when taking magnesium than when not.

Enrolling patients with depression listed on their medical chart resulted in missing people with undiagnosed depression or who do not use Primary Care. PCPs may have introduced selection bias by differentially disapproving patients they thought were unlikely to be open to alternative treatments. This may not be an important limitation to generalizability since nutraceuticals would probably not be recommended for these patients anyway. The low response rate to our letter of invitation and follow up calls may have also introduced selection bias.

The study excluded subjects with malabsorption because the main known side effect of magnesium is diarrhea. However, because diarrhea was rare in the study, it would be worth determining the tolerance and effect in those with GI disease. Some of the subgroups are small, limiting our ability to detect variation in efficacy, although none was seen. Due to the makeup of the local community, the study population lacked racial diversity.

Although improvement in symptoms occurred within two weeks and was maintained while on treatment, long-term effectiveness is unknown and longer trials are needed.

Measurement of serum magnesium was outside the scope of the study. A recent meta-analysis of observational studies found an overall 1.3-fold increased risk of depression in people with hypomagnesaemia [37] yet a previous met-analysis was inconclusive [38]. It is not clear if hypomagnesaemia influences the efficacy of magnesium supplementation for depression.

## Conclusions

Daily supplementation with 248 mg of elemental magnesium as four 500 mg tablets of magnesium chloride per day leads to a significant decrease in depression and anxiety symptoms regardless of age, gender, baseline severity of depression, or use of antidepressant medications. While the cross over design of this trial is robust in controlling for internal biases, it would be reassuring to see the results replicated in larger clinical trials that test long term efficacy and provide additional data on subgroups. However, this efficacy trial showed magnesium supplements may be a fast, safe, and easily accessible alternative, or adjunct, to starting or increasing the dose of antidepressant medications.

## Supporting information

S1 File Consort checklist. (DOC)

S2 File Study protocol. (PDF)

S3 File Insituttional Review Board approval. (PDF)

S4 File Data for all contacted participants. (XLSX)

S5 File Data for randomized participants. (XLSX)
