# Supplementary material for: Role of magnesium supplementation in the treatment of depression: A randomized clinical trial
Source: PLoS One. 2017 Jun 27;12(6):e0180067. doi: 10.1371/journal.pone.0180067 (PMC5487054; doi:10.1371/journal.pone.0180067)
Supplement: S2 File — (PDF) [file pone.0180067.s002.pdf]

The University of Vermont Committees on Human Research

For Committee Use Only
PROTOCOL NUMBER

Human Subjects Research Protocol

The Common Human Subjects Protocol Cover Form **must** be completed and **attached** to the front of this form. This Protocol form should be completed for any human subjects research proposal that does not have a specific "protocol," such as a grant application. This form must be submitted along with a copy of the complete grant proposal and all the information in this form **must** be consistent with that proposal. This protocol form, once IRB approved, will be the working protocol for that research. **When completing this document, do not refer to page numbers within your grant**. If revisions are necessary during the course of the research, amendments should refer to this protocol form, <u>not</u> the grant proposal. Enter responses for all sections. Check N/A if the section does not apply.

PROTOCOL SUMMARY

Project: Title (Should match the title entered on the face page of any associated grant proposal.)

Role of Magnesium Supplementation in the Treatment of Depression

Principal Investigator: Emily Tarleton, MS, RD

Grant Sponsor:

Grant Number:
(For grants routed through UVM, indicate the OSP Proposal ID # located at the top of the OSP Routing Form)

Lay Language Summary: *(Please use <u>non-technical</u> language that would be understood by nonscientific IRB members to summarize the proposed research project. The information must include: (1) a brief statement of the problem and related theory supporting the intent of the study, and (2) a brief but specific description of the procedure(s) involving the human subjects. Please do not exceed one single-spaced 8 ½ X 11" page.)*

Depression is a common and disabling disorder. Almost 11% of adults older than 60 and 18.8% of those younger than 60 suffer from depression (Centers for Disease Control and Prevention 2010). Although both pharmacologic and behavioral therapies are effective for many patients, they have important limitations. Medications can take weeks to have an effect, often have significant adverse effects, and fail to help many patients at all (Zarate, Duman et al. 2013). Non-pharmacologic approaches such as Cognitive Behavioral Therapy are also effective (Hoifodt, Strom et al. 2011), but require highly trained therapists and weeks to achieve effectiveness.

As a result of the need for additional treatment options, interest in the role of nutrition in modulating depressive symptoms has grown. Magnesium plays a role in many of the pathways involved in the pathophysiology of depression and is found in several enzymes, hormones, and neurotransmitters (Serefko, Szopa et al. 2013). Depression and magnesium are both associated with systemic inflammation (Chacko, Sul et al. 2011, Maes 2011). National data indicate a majority of the population has magnesium intake below the Recommended Daily Allowance (Ford and Mokdad 2003).

Magnesium supplementation has been linked to improvement in symptoms of major depression (Eby and Eby 2006), premenstrual symptoms (Walker, De Souza et al. 1998), postpartum depression (Eby and Eby 2006) and chronic fatigue syndrome (Cox and Campbell 1991). Low magnesium status has been associated with increased depressive symptoms in several different age groups and ethnic populations (Barragan-Rodriguez, Rodriguez-Moran et al. 2008, Jacka, Overland et al. 2009, Jacka, Maes et al. 2012, Yary, Aazami et al. 2013). Issues in study design have led to inconclusive results and skepticism of magnesium's role in depression. With varying outcomes, different populations and age ranges, and limited sample sizes, consensus on the relationship between magnesium intake and depression has not been reached.

This study will utilize the Patient Health Questionnaire 9 (Kroenke, Spitzer et al. 2001) and General Anxiety Disorder 7 Item (Spitzer, Kroenke et al. 2006) questionnaires to determine changes in depressive symptoms and anxiety over a period of 12 weeks. Magnesium supplements (Alta Health 64mg Mag Chloride tablets http://www.altahealthproducts.com/mag.html) will be provided for 6 of the 12 weeks. Volunteers will be randomly assigned to start the supplements at week 1 or at week 7. Biweekly calls to the volunteer will allow for monitoring of side effects, changes in health or prescription medication use, and monitoring of depressive symptoms.

PURPOSE AND OBJECTIVES

Purpose: *The importance of the research and the potential knowledge to be gained should be explained in detail. Give background information.*

The purpose of this study is to test the hypothesis that magnesium supplementation decreases depressive symptoms. The primary outcome measure is the PHQ-9 questionnaire, a validated measure of depression. Secondary measures include the GAD-7 for Anxiety, side effects, and intention to continue supplementation outside of the study.

If proven effective, magnesium supplementation might address some of the limitations of currently available treatment for depression. Treatment with magnesium might be attractive to patients that have previously experienced unwanted side effects from medications for depression. Although it can lead to hypermagnesemia and diarrhea, magnesium is, in general, a safe treatment with few unanticipated side effects. Supplementation with magnesium has shown very quick results. Case studies of magnesium supplementation reported improvements in depression, anxiety, and sleep within one week (Eby and Eby 2006, Barragan-Rodriguez, Rodriguez-Moran et al. 2008).

References*. Include references to prior human or animal research and references that are relevant to the design and conduct of the study.*

Barragan-Rodriguez, L., M. Rodriguez-Moran and F. Guerrero-Romero (2008). "Efficacy and safety of oral magnesium supplementation in the treatment of depression in the elderly with type 2 diabetes: a randomized, equivalent trial." Magnes Res **21**(4): 218-223.

Centers for Disease Control and Prevention (2010). "Current Depression Among Adults---United States, 2006 and 2008." Morbidity and Mortality Weekly Update **59**(38): 1229-1235.

Chacko, S. A., J. Sul, Y. Song, X. Li, J. LeBlanc, Y. You, A. Butch and S. Liu (2011). "Magnesium supplementation, metabolic and inflammatory markers, and global genomic and proteomic profiling: a randomized, double-blind, controlled, crossover trial in overweight individuals." Am J Clin Nutr **93**(2): 463-473.

Cox, I. M. and M. J. Campbell (1991). "Red blood cell magnesium and chronic fatigue syndrome." Lancet **337**(8744): 757.

Eby, G. A. and K. L. Eby (2006). "Rapid recovery from major depression using magnesium treatment." Medical Hypotheses **67**(2): 362-370.

Ford, E. S. and A. H. Mokdad (2003). "Dietary magnesium intake in a national sample of US adults." J Nutr **133**(9): 2879-2882.

Hoifodt, R. S., C. Strom, N. Kolstrup, M. Eisemann and K. Waterloo (2011). "Effectiveness of cognitive behavioural therapy in primary health care: a review." Fam Pract **28**(5): 489-504.

Jacka, F. N., M. Maes, J. A. Pasco, L. J. Williams and M. Berk (2012). "Nutrient intakes and the common mental disorders in women." J Affect Disord **141**(1): 79-85.

Jacka, F. N., S. Overland, R. Stewart, G. S. Tell, I. Bjelland and A. Mykletun (2009). "Association between magnesium intake and depression and anxiety in community-dwelling adults: the Hordaland Health Study." Aust N Z J Psychiatry **43**(1): 45-52.

Kroenke, K., R. Spitzer and J. Williams (2001). "The PHQ-9: validity of a brief depression severity measure." J Gen Intern Med **16**: 606 - 613.

Littenberg, B. and C. MacLean (2006). "Intra-cluster correlation coefficients in adults with diabetes in primary care practices: the Vermont Diabetes Information System field survey." BMC Medical Research Methodology **6**(1): 20.

Maes, M. (2011). "Depression is an inflammatory disease, but cell-mediated immune activation is the key component of depression." Prog Neuropsychopharmacol Biol Psychiatry **35**(3): 664-675.

Morisky, D. E., A. Ang, M. Krousel-Wood and H. J. Ward (2008). "Predictive Validity of a Medication Adherence Measure in an Outpatient Setting." The Journal of Clinical Hypertension **10**(5): 348-354.

Pinto-Meza, A., A. Serrano-Blanco, M. T. Peñarrubia, E. Blanco and J. M. Haro (2005). "Assessing Depression in Primary Care with the PHQ-9: Can It Be Carried Out over the Telephone?" Journal of General Internal Medicine **20**(8): 738-742.

Serefko, A., A. Szopa, P. Wlaz, G. Nowak, M. Radziwon-Zaleska, M. Skalski and E. Poleszak (2013). "Magnesium in depression." Pharmacol Rep **65**(3): 547-554.

Spitzer, R. L., K. Kroenke, J. W. Williams and B. Löwe (2006). "A brief measure for assessing

generalized anxiety disorder: The gad-7." Archives of Internal Medicine **166**(10): 1092-1097.
Walker, A. F., M. C. De Souza, M. F. Vickers, S. Abeyasekera, M. L. Collins and L. A. Trinca (1998). "Magnesium supplementation alleviates premenstrual symptoms of fluid retention." J Womens Health **7**(9): 1157-1165.
Yary, T., S. Aazami and K. Soleimannejad (2013). "Dietary intake of magnesium may modulate depression." Biol Trace Elem Res **151**(3): 324-329.
Zarate, C., R. S. Duman, G. Liu, S. Sartori, J. Quiroz and H. Murck (2013). "New paradigms for treatment-resistant depression." Annals of the New York Academy of Sciences **1292**(1): 21-31.

Objectives: *Clearly state the primary and secondary objective(s) of the study.*

The primary objective is to determine whether supplementation with oral magnesium significantly changes PHQ-9 scores. Secondary objectives include whether magnesium supplementation changes GAD-7 scores, to track side effects, and evaluate efficacy of supplements from the volunteer's perspective.

METHODS AND PROCEDURES

Study Design: *Describe the research design, including a description of any new methodology and its advantage over existing methodologies.*

We will conduct a randomized, crossover study.

Procedures: *Describe all procedures (sequentially) to which human participants will be subjected. Identify all procedures that are considered experimental and/or procedures performed exclusively for research purposes. Describe the types, frequency and duration of tests, study visits, interviews, questionnaires, etc. Include required screening procedures performed before enrollment and while on study. Please provide in table, list or outline format for ease of review. (describe and attach all instruments)*

Note: A clinical research protocol may involve interventions that are strictly experimental or it may involve some aspect of research (e.g., randomization among standard treatments for collection and analysis of routine clinical data for research purposes). It is important for this section to distinguish between interventions that are experimental and/or carried out for research purposes versus those procedures that are considered standard therapy. In addition, routine procedures performed solely for research purposes (e.g., additional diagnostic/follow-up tests) should be identified.

This study takes place over 12 consecutive weeks. While we will follow the volunteers for the full 12 weeks they will only take magnesium supplements for 6 consecutive weeks. They will be randomly assigned to start the supplement at week 1 or week 7. They will take two Alta Health supplement two times a day for a total of 248 mg elemental magnesium. This amount of magnesium is less than the tolerable upper limit of 350 mg per day. The supplements will be provided. Volunteers will be asked to maintain their normal diet for the 12 weeks of the study.

What is expected of the volunteers throughout the study?

- Complete a baseline interview at home or another location convenient for the volunteer.
- Take the supplements twice daily for either the first or second 6 weeks of the study period.
- Complete brief telephone interviews every 2 weeks to include the PHQ-9 and GAD-7 questionnaires.
- Inform the PI if they become pregnant during the study.
- No clinic visits, blood draws, or other invasive data collection are required.

At the final call the volunteers will be asked to count out the remaining pills.

Below is a table showing the timeline of the study and what will happen each week.

Week	Baseline	1	2	3	4	5	6	7	8	9	10	11	12
Questionnaire	x												
PHQ-9	x		x		x		x		x		x		x
GAD-7	x		x		x		x		x		x		x
phone call			x		x		x		x		x		x
Supplements Provided	x*							x*					

* Supplements provided either at baseline or week 7.

We will provide written volunteer instructions (attached) with detailed instructions for taking the supplements. The biweekly phone call questions will vary depending on randomization (attached as part of the "Volunteer Record"). The last phone call

while the volunteers are taking the supplements will include a final pill count to determine percent compliance. If the supplements are taken according to the protocol they should have 32 left over. In addition we will use the Modified Morisky Scale to measure adherence (Morisky, Ang et al. 2008).

For research involving survey, questionnaires, etc.: *Describe the setting and the mode of administering the instrument and the provisions for maintaining privacy and confidentiality. Include the duration, intervals of administration, and overall length of participation. (describe and attach all instruments)*

☐ ***Not applicable***

The initial PHQ-9 and GAD-7questionnaires will be done over the phone to determine eligibility. Subsequent questionnaires will be completed over the phone during the biweekly calls. A validation study has shown similar PHQ-9 results over the phone versus in person (Pinto-Meza, Serrano-Blanco et al. 2005). Completed questionnaires will be kept in a locked drawer and entered into a database on a secure network. The Modified Morisky Scale will be completed over the phone at the final call. Each questionnaire takes about 5-10 minutes to complete.

Statistical Considerations: *Delineate the precise outcomes to be measured and analyzed. Describe how these results will be measured and statistically analyzed. Delineate methods used to estimate the required number of subjects. Describe power calculations if the study involves comparisons. Perform this analysis on each of the primary and secondary objectives, if possible.*

Descriptive statistics will be used to describe the population. T-tests will determine changes in PHQ-9 and GAD-7 scores while on magnesium vs. not. Linear regression will be used to control for confounders and when the supplement was given during the study timeline. We will use the intention to treat analysis.

Microsoft excel will be used to develop a randomization table in blocks of 10. We will transfer the randomization information to an opaque envelope and seal it. Ten slips of paper will be put in 10 envelopes to start (for a total of 100 randomization slots). Once the envelopes are sealed they will be shuffled and then labeled with numbers 1-10. The envelopes will be opened in numerical order and volunteers will be randomized in accordance to the next available slot on the 10 block randomization in the envelope. Additional blocks of 10 will be generated and more envelopes created as needed to account for drop outs.

Confidentiality Measures and Secure Storage of Data or Tissue: *Describe how the data/tissue will be collected. Will there be identifiers or will the data/tissue be coded? Describe where the data/tissue will be stored and how it will be secured. Describe who will have access to the data/tissue or the codes. If subject data/tissues with identifiers will be released, specify to whom. Describe what will happen to the data/tissues when the research has been completed.*

☐ ***Not Applicable***

The PI will assign a study number to the subject. That subject number will go on the questionnaires and only the study number will be entered into a database. Answers to the questionnaires, collected data, and the information on randomization will be kept in the database. The database is on a secured UVM Medical Center database (CTS department). Original copies of the questionnaires and logs will be stored in a locked drawer in the PIs office on MCHV Campus of the UVM Medical Center.

Risks/Benefits: *Describe any potential or known risks. This includes physical, psychological, social, legal or other risks. Estimate the probability that given risk may occur, its severity and potential reversibility. If the study involves a placebo or washout period, the risks related to these must be addressed in both the protocol and consent. Describe the planned procedures for protecting against or minimizing potential risks and assess their likely effectiveness. Where appropriate, discuss plans for ensuring necessary medical or professional intervention in the event of adverse effects to the subjects. Discuss the potential benefits of the research to the subjects and others. Discuss why the risks to the subjects are reasonable in relation to the anticipated benefits to subjects and others. Discuss the importance of the knowledge gained or to be gained as a result of the proposed research and why the risks are reasonable in relation to the knowledge that reasonably may result. If there are no benefits state so.*

Potential Risks:
The dose of magnesium for this study is less than the tolerable upper limit of 350 mg elemental magnesium per day and therefore the risk to volunteers is low, including risk of toxicity.

By excluding people with renal insufficiency (glomerular filtration rate <60) we will avoid adverse effects secondary to inability to clear magnesium from the body. Volunteers will be called weekly and asked about adverse events. Nausea and diarrhea are the most common side effects. Most of the time, nausea and diarrhea will resolve over a few days and severe side effects can be avoided. Volunteers will be instructed to take only 1 or 2 tablet per day for 2-3 days if diarrhea or nausea persists and to contact the PI. If they feel the need to stop the supplements because of side effects they will be instructed to do so and to contact the PI. In addition if they are experiencing dizziness when standing, profuse watery stools, or have stopped making urine they should stop the supplements and seek immediate medical treatment.

We are excluding people with severe depression (PHQ-9 score ≥20) but we will have a suicide action plan in place. Benjamin Littenberg, MD will be called to speak to the patient in the event that unanticipated problems arise. Worsening depression, as indicated by a 10 point increase in PHQ-9 score or a positive answer to question #9 ("Thoughts that you would be better off dead, or of hurting yourself in some way") will result in instructions to stop the supplements, see their physician immediately and/or go to the local emergency department or call the national suicide hotline for immediate help.
Participation does involve the potential risks of a breach of confidentiality and associated privacy of the participants. Such risks will be minimized by 1) securing, in a separate location, and limiting access to information with direct participant identifiers; and 2) limiting access to information collected to the research team

Other unanticipated side effects will be recorded and tracked biweekly.

Potential Benefits:
If our hypothesis is correct, there is the potential benefit of improvement in depressive symptoms.

Therapeutic Alternatives: *List the therapeutic alternatives that are reasonably available that may be of benefit to the potential subject and include in the consent form as well.*

☐ ***Not Applicable***

Prescription medications for depression are available. If subjects are currently on medication for depression, this study will be offered in conjunction with medication since they must still score a 5 or above on the PHQ-9 (but not greater than 19), indicating at least some level of depression even with medication. If the potential volunteer scores ≥5 on the PHQ-9 and is not on medication, they will be told about their options for treatment, including medication and therapy. If they choose medication or therapy they will not be eligible for this study until treatment has been stable for at least 2 months.

Data Safety and Monitoring: *The specific design of a Data and Safety Monitoring Plan (DSMP) for a protocol may vary extensively depending on the potential risks, size, and complexity of the research study. For a minimal risk study, a DSMP could be as simple as a description of the Principal Investigator's plan for monitoring the data and performance of safety reviews or it could be as complex as the initiation of an external, independent Data Safety and Monitoring Board (DSMB). The UVM/UVM MEDICAL CENTER process for review of adverse events should be included in the DSMP.*

The data will be stored on a secure UVM Medical Center server, specifically within the S Drive Clinical and Translational Science Department sub directory. The data and safety monitoring plan will involve routine (i.e., annually) monitoring by the Principal Investigator of any conditions that may negatively impact the confidentiality of information. Any unauthorized access to medical record information or linking of information to participant direct identifiers shall be reported to the IRB.

Adverse Event and Unanticipated Problem (UAP) Reporting: *Describe how events and UAPs will be evaluated and reported to the IRB. All protocols should specify that, in the absence of more stringent reporting requirements, the guidelines established in the Committees on Human Research "Adverse Event and Unanticipated Problems Reporting Policy" will be followed. The UVM/UVM MEDICAL CENTER process for review of adverse events and UAPs to subjects or others should be included in the DSMP.*

The guidelines established in the Committees on Human Research "Adverse Event and Unanticipated Problems Reporting Policy" will be followed.

Withdrawal Procedures: *Define the precise criteria for withdrawing subjects from the study. Include a description of study requirements for when a subject withdraws him or herself from the study (if applicable).*

Subjects can withdraw him or herself at any time but the data already collected may still be used in analysis. Because we are conducting an intention to treat analysis, all subjects will be included in the final analysis. Volunteers with worsening depression, as indicated by a 10 point increase in PHQ-9 score or a positive answer to question #9 ("Thoughts that you would be better off dead, or of hurting yourself in some way") will stop the supplements and no longer be included in the study.

Sources of Materials: *Identify sources of research material obtained from individually identifiable human subjects in the form of specimens, records or data. Indicate whether the material or data will be obtained specifically for research purposes or whether use will be made of existing specimens, records or data.*

The PHQ-9 and GAD-7 (attached) are both validated questionnaires used regularly in the primary care setting as quick and accurate assessments of current symptoms. These questionnaires are for research purposes only.

DRUG AND DEVICE INFORMATION

Investigators are encouraged to consult the UVM MEDICAL CENTER Investigational Pharmacy Drug Service (847-4863) prior to finalizing study drug/substance procedures.

Drug (s) ☐ ***Not applicable***

Drug name – generic followed by brand name and common abbreviations. Availability – Source and pharmacology; vial or product sizes and supplier. If a placebo will be used, identify its contents and source. (attach investigational drug brochure)

Alta Health Magnesium chloride is an over the counter supplement. Volunteers will receive the original, sealed bottles (containing 100 tablets) of the magnesium that have been purchased for them. Lot numbers and expiration dates will be logged as well as when and to whom it was given. We will keep a "Magnesium Accountability Form" (attached) modeled after the NCI accountability form http://ctep.cancer.gov/forms/docs/oral_agent_accountability.pdf. *I have met with IDS and discussed storage, dosage, documentation, and accountability.*

Preparation: Reconstitution instructions; preparation of a sterile product, compounded dosage form; mixing guidelines, including fluid and volume required. Identify who will prepare.

Magnesium Chloride– two tablets twice a day with water for a total of 248 mg elemental magnesium.

Storage and stability – for both intact and mixed products.

Before being given to volunteers, it will be stored in a room with a temperature control (70 degrees Fahrenheit). Volunteers will be told to store supplements at room temperature in a dry location (see Instructions for Volunteers).

Administration – Describe acceptable routes and methods of administration and any associated risks of administration.

Oral administration

Toxicity – Accurate but concise listings of major toxicities. Rare toxicities, which may be severe, should be included by indicated incidence. Also adverse interactions with other drugs used in the protocol regimen as well as specific foods should be noted. Address significant drug or drug/food interactions in the consent form as well. List all with above details.

According to the NIH very large doses of magnesium-containing and antacids (typically providing more than 5,000 mg/day magnesium) have been associated with magnesium toxicity. We are providing only 5% of this amount and it is less than the

tolerable upper limit of 350 mg per day. http://ods.od.nih.gov/factsheets/Magnesium-HealthProfessional/

People with renal insufficiency are at higher risk for toxicity so we have excluded this group.

Is it FDA approved: (include FDA IND Number)

1. *in the dosage form specified? If no, provide justification for proposed use and source of the study drug in that form.*

No. Supplements do not go through the same FDA approval process as drugs. Previous studies in the U.S. have used magnesium in other forms (not chloride) for clinical trials. Magnesium chloride is better absorbed, which is why we chose this form. The RDA for women >18 years is 360 mg/d and for men >18 years 410 mg/d. Each tablet contains: 62.17mg elemental Magnesium Chloride (per the manufacturer http://www.altahealthproducts.com/mag.html). The elemental magnesium in the dose we are giving (4 tablets) is below the upper tolerable limit.

2. *for the route of administration specified? If no, provide justification for route and describe the method to accomplish.*

3. *for the intended action?*

No, an IND application has been submitted and we are waiting for a response from the FDA.

Device (s) [x] ***Not applicable***

Device name and indications (attach investigational device brochure)

Is it FDA approved: (include FDA IDE Number)

1. *for indication specified? If no, provide justification for proposed use and source of the device.*

Risk assessment (non-significant/significant risk) - PI or sponsor needs to assess risk of a device based upon the use of the device with human subjects in a research environment.

SUBJECT CHARACTERISTICS, IDENTIFICATION AND RECRUITMENT

Subject Selection: *Provide rationale for subject selection in terms of the scientific objectives and proposed study design.*

We will recruit adults (age ≥18 years old) who have a documented Glomerular Filtration Rate of >60 in the past year and have depression in their problem list. Subsequently, only people with an initial PHQ-9 score between 5 and 19 will be invited to participate (mild to moderate depression). People who are planning to start or change therapy or medications for depression will be asked to contact us again once they have been on stable treatment for at least 2 months.

Vulnerable Populations: *Explain the rationale for involvement of special classes of subjects, if any. Discuss what procedures or practices will be used in the protocol to minimize their susceptibility to undue influences and unnecessary risk (physical, psychological, etc.).*

[x] ***Not applicable***

Number of Subjects: *What is the anticipated number of subjects to be enrolled at UVM/UVM MEDICAL CENTER and in the case of a multi-center study, with UVM/UVM MEDICAL CENTER as the lead, the total number of subjects for the entire study.*

100 - α=0.05, β=0.844, μ=1.5 (clinically significant change in PHQ-9), and SD=5 (Littenberg and MacLean 2006).

Inclusion/Exclusion Criteria: *Eligibility and ineligibility criteria should be specific. Describe how eligibility will be determined and by whom. Changes to the eligibility criteria at a later phase of the research have the potential to invalidate the research.*

Eligibility will be determined by the PI (with consultation with Benjamin Littenberg, MD as needed) after the volunteer has signed the consent form and allowed access to their medical record.

Inclusion:

- All adults at least 18 years of age
- A Patient Health Questionnaire-9 (PHQ-9) score of greater or equal to 5 but less than 20
- People who are currently being treated for depression are still eligible to participate but their treatment must be stable (no changes in medication dose or brand and/or no changes in therapy regimen for at least 2 months).

Exclusion:

- Active delirium or dementia
- Medicinal treatment for bipolar disorder, personality disorder or schizophrenia,
- Glomerular Filtration Rate of less than 60
- Irritable Bowel Disease
- Inflammatory Bowel Disease
- GERD
- Gastritis
- Pregnant as reported by potential volunteer
- Myasthenia Gravis
- Planned elective surgery
- Currently taking
  - Long Term Antibiotics
  - Fluoroquinolone
  - Trientine or Penicillamine
  - Long Term Antivirals

- Digoxin
- Bisphosphonates
- Eltrombopag
- Opiods
- Calcium Channel Blockers
- Deferiprone
- Doxercalciferol

- Unable or unwilling to stop taking a MVI or magnesium supplement

Due to the complexity of state and federal requirements governing the participation of prisoners in research, prisoners-patients shall not be approached for participation.

Inclusion of Minorities and Women: *Describe efforts to include minorities and women. If either minorities or women are excluded, include a justification for the exclusion.*

The racial, gender and ethnic characteristics of the individuals approached for participation in this study shall reflect the demographics of the area of the country in which they live. We shall attempt to recruit participants in accordance with these demographics. No individuals shall be excluded from participation based on race, ethnicity, or gender.

Inclusion of Children: *Describe efforts to include children. Inclusion is required unless a clear and compelling rationale shows that inclusion is inappropriate with respect to the health of the subjects or that inclusion is inappropriate for the purpose of the study. If children are included, the description of the plan should include a rationale for selecting or excluding a specific age range of children. When included, the plan must also describe the expertise of the investigative team in working with children, the appropriateness of the available facilities to accommodate children, and the inclusion of a sufficient number of children to contribute to a meaningful analysis relative to the purpose of the study.* ***If children are excluded*** *then provide appropriate justification. Provide target accrual for this population.*

We will exclude anyone under the age of 18. Previous studies using magnesium supplementation have focused on adults. At this time, it is not clear what a safe dose of magnesium is for children and what side effects might occur.

For protocols including the use of an investigational drug, indicate whether women of childbearing potential have been included and, if not, include appropriate justification.

We are excluding women who are pregnant because magnesium supplementation has not been proven to be safe during pregnancy according to the American Academy of Pediatrics.

If HIV testing is included specifically for research purposes explain how the test results will be protected against unauthorized disclosure. Include if the subjects are to be informed of the test results. If yes, include the process and provision for counseling. If no, a rationale for not informing the subjects should be included.

x	***Not applicable***

Recruitment: *Describe plans for identifying and recruitment of subjects. All recruitment materials (flyers, ads, letters, etc) need to be IRB approved prior to use.*

Primary care providers will be invited to identify potentially eligible subjects by screening the records of adults in their practice meeting the following criteria: depression on the problem list, Glomerular Filtration Rate (GFR) of >60 documented in the past year, absence of GERD or gastritis, and the absence of certain medications (Long Term Antibiotics, Trientine or Penicillamine, Long Term Antivirals, Digoxin, Bisphosphonates, Eltrombopag, Opiods, Calcium Channel Blockers, Deferiprone, Doxercalciferol). PCPs will be asked to review the data from their practices themselves. If this is not practicable (as for most UVMMG PCPs), Dr. MacLean will identify potentially eligible subjects from the research data set created under protocol 15-089 “Health Services Research and Pharmacoepidemiology at Fletcher Allen Health Care” and share the list with the PCP. For each potentially eligible patient approved by the PCP, the research team will send a letter of invitation on behalf of the PCP on the PCPs letterhead (see Patient Recruitment Letter). The research team will follow-up by telephone to confirm interest. See “Study Intro Call” script attached. If the patient is interested we will request verbal consent to ask initial screening and eligibility questions (PHQ-9, GAD-7, medication use, pregnancy status, history of bowel disease). Once eligibility is determined, we will schedule consent and enrollment as appropriate. See “Consent Process” diagram attached.

FINANCIAL CONSIDERATIONS

Expense to Subject: *If the investigation involves the possibility of added expense to the subject (longer hospitalization, extra studies, etc.) indicate in detail how this will be handled. In cases where the FDA has authorized the drug or device company to charge the patient for the experimental drug or device,* ***a copy of the authorization letter from the FDA or sponsor must accompany the application. Final approval will not be granted until the IRB receives this documentation.***
There are very limited circumstances under which study participants may be responsible (either directly or via their insurance) for covering some study-related expenses. If the study participant or their insurer(s) will be billed for any portion of the research study, provide a justification as to why this is appropriate and acceptable. For example, if the study involves treatment that is documented standard of care and not investigational, state so. In these cases, the protocol and the consent should clearly define what is standard of care and what is research.

There is no expense to the subject. All supplements will be provided to the subjects.

Payment for participation: *Describe all plans to pay subjects, either in cash, a gift or gift certificate. Please note that all payments must be prorated throughout the life of the study. The IRB will not approve a study where there is only a lump sum payment at the end of the study because this can be considered coercive. The amount of payment must be justified. Clarify if subjects will be reimbursed for travel or other expenses.*

x	***Not applicable***

***Collaborating Sites**. When research involving human subjects will take place at collaborating sites or other performance sites when UVM/UVM MEDICAL CENTER is the lead site, the principal investigator must provide in this section a list of the collaborating sites and their Federalwide Assurance numbers when applicable. (agreements may be necessary)*

x	***Not applicable***

INFORMED CONSENT

***Consent Procedures**: Describe the consent procedures to be followed, including the circumstances under which consent will be obtained, who will seek it, and the methods of documenting consent.*

Note: Only those individuals authorized to solicit consent may sign the consent form confirming that the prospective subject was provided the necessary information and that any questions asked were answered.

People who express interest in participating will be given the PHQ-9 and GAD-7 over the phone. If they score between 5 and 19 on the PHQ-9 they will be invited to participate and the PI will set up a meeting to go over the consent form. The PI will meet the volunteer either at the UVM/UVM Medical Center Campus or somewhere convenient for the volunteer to sign the consent form. The Volunteer will be able to take the form home and given as much as they would like to review it and decide whether they would like to sign it.

***Information Withheld From Subjects:** Will any information about the research purpose and design be withheld from potential or participating subjects? If so, explain and justify the non-disclosure and describe plans for post-study debriefing.*

x	***Not applicable***

***Consent, Assent, and HIPAA Authorization.** Specify the form(s) that will be used e.g. consent (if multiple forms explain and place identifier on each form), assent form and/or HIPAA authorization (if PHI is included). These form(s) must accompany the protocol as an appendix or attachment.*

The consent form and HIPAA Authorization will both be signed before randomization.

Attach full grant application, including budget information and/or any contract or draft contract associated with this application.
