# Supplementary material for: Role of magnesium supplementation in the treatment of depression: A randomized clinical trial
Source: PLoS One. 2017 Jun 27;12(6):e0180067. doi: 10.1371/journal.pone.0180067 (PMC5487054; doi:10.1371/journal.pone.0180067)
Supplement: S3 File — (PDF) [file pone.0180067.s003.pdf]

The UNIVERSITY of VERMONT
Committees on Human Subjects
Serving the University of Vermont
and the UVM Medical Center

RESEARCH PROTECTIONS OFFICE
213 Waterman Building
85 South Prospect Street
Burlington, Vermont 05405
(802)656-5040 ph
www.uvm.edu/irb/

CHRMS: 15-337

Protection of Human Subjects Assurance

Title: Role of Magnesium Supplementation in the Treatment of Depression

Principal Investigator: Emily Tarleton

Institution: University of Vermont and State Agricultural College, Burlington, VT 05405

This institution has an approved assurance of compliance on file with the Department of Health and Human Services which covers this activity.

University of Vermont and State Agricultural College: FWA 00000723 Expiration Date: Nov 12, 2018

The UVM Medical Center: FWA 00000727 Expiration Date: November 12, 2019

IRB number 00000485

Certification of IRB Review

This activity has been reviewed and approved by an IRB in accordance with the requirements of 45 CFR 46, including its relevant Subparts; and, when applicable, with the requirements of 21 CFR 50 and 21 CFR 56.

Date of approval APR 15 2015 Date of expiration MAR 16 2016

IRB Review Type: Full review

Institutional Signature/Date: [signature] 4.15.15

Name and Title of Official: Deborah Z. Rubin, M.D., Chair,
Committee on Human Research in the Medical Sciences
